# Microglia activation visualization via fluorescence lifetime imaging microscopy of intrinsically fluorescent metabolic cofactors

**DOI:** 10.1117/1.NPh.7.3.035003

**Published:** 2020-08-08

**Authors:** Md. Abdul K. Sagar, Jonathan N. Ouellette, Kevin P. Cheng, Justin C. Williams, Jyoti J. Watters, Kevin W. Eliceiri

**Affiliations:** a University of Wisconsin-Madison, Department of Biomedical Engineering, Madison, Wisconsin, United States; b University of Wisconsin-Madison, Department of Comparative Biosciences, Madison, Wisconsin, United States; c University of Wisconsin-Madison, Department of Medical Physics, Madison, Wisconsin, United States; d Morgridge Institute for Research, Madison, Wisconsin, United States

**Keywords:** microglia, activation, lipopolysaccharide, noninvasive, nicotinamide adenine dinucleotide, fluorescence lifetime imaging microscopy

## Abstract

**Significance:** A major obstacle to studying resident microglia has been their similarity to infiltrating immune cell types and the lack of unique protein markers for identifying the functional state. Given the role of microglia in all neural diseases and insults, accurate tools for detecting their function beyond morphologic alterations are necessary.

**Aims:** We hypothesized that microglia would have unique metabolic fluxes in reduced nicotinamide adenine dinucleotide (NADH) that would be detectable by relative changes in fluorescence lifetime imaging microscopy (FLIM) parameters, allowing for identification of their activation status. Fluorescence lifetime of NADH has been previously demonstrated to show differences in metabolic fluxes.

**Approach:** Here, we investigate the use of the label-free method of FLIM-based detection of the endogenous metabolic cofactor NADH to identify microglia and characterize their activation status. To test whether microglial activation would also confer a unique NADH lifetime signature, murine primary microglial cultures and adult mice were treated with lipopolysaccharide (LPS).

**Results:** We found that LPS-induced microglia activation correlates with detected changes in NADH lifetime and its free-bound ratio. This indicates that NADH lifetime can be used to monitor microglia activation in a label-free fashion. Moreover, we found that there is an LPS dose-dependent change associated with reactive microglia lifetime fluxes, which is also replicated over time after LPS treatment.

**Conclusion:** We have demonstrated a label-free way of monitoring microglia activation via quantifying lifetime of endogenous metabolic coenzyme NADH. Upon LPS-induced activation, there is a significant change in the fluorescence lifetime following activation. Together, these results indicate that NADH FLIM approaches can be used as a method to characterize microglia activation state, both *in vitro* and *ex vivo*.

## 1 Introduction

Microglia have prominent roles in both central nervous system (CNS) injury/disease responses and in protection of the CNS.^1^^–^^4^ Major obstacles in studying microglia have been the lack of specific protein markers that indicate their activation status in the absence of concomitant morphological changes and the difficulty in distinguishing them from related infiltrating myeloid cells. Microglial activation can be characterized in many different ways, but currently there is no single parameter that can uniquely characterize their activation,^5^ likely because the process of activation is gradual and involves many intermediate states on the spectrum between surveillance and activation.^6^ While proteins such as ionized calcium binding protein 1 (Iba1), the integrin receptor CD11b, the purinergic receptor P2Y12, and the fractalkine receptor CX3CR1 are uniquely expressed in microglia in the healthy CNS,^7^^,^^8^ they are also expressed in macrophages, preventing accurate distinction between resident microglia and infiltrating peripheral cells. The current primary method of observing microglial activation depends on antibody staining to assess morphology and marker expression. For example, $\mathrm{CD}68^{\text{high}}$ immunoreactivity detects both activated microglia and macrophages. While the combination of $\mathrm{CD}11^{b+}$ and $\mathrm{CD}45^{\text{low}}$ staining is widely used to distinguish microglia from macrophages, it cannot distinguish between activated microglia and macrophages. More recently, other proteins such as the orphan receptor TMEM119^9^ and the lysosomal hexosaminidase enzyme B (HexB)^10^ have been identified by transcriptomic analyses as unique to microglia, and are not expressed in macrophages,^11^^–^^14^ but these cannot distinguish activation states either. Thus, a method allowing for a clear distinction in microglial activation states would be indispensable for identifying initiators of and/or contributors to CNS dysfunction in different disease etiologies. To study microglial activity *ex vivo*, methods such as immunomagnetic separation can efficiently isolate microglia without altering their functional state.^15^ However, a method that does not require isolation from the rest of the CNS environment that can enable analysis of microglial activation status in intact tissue or whole brain would be more powerful. Recently, morphology-based image analysis techniques have been developed to characterize microglial activation based on the ratio of cell body to cell size,^16^ or morphological categorization based on hierarchical cluster and principal components analysis.^17^ Other studies have tried to characterize microglial activation using statistical changes in soma size and roundness.^18^

To address the limitations of current antibody-based microglial characterization techniques, we hypothesized that fluorescence lifetime imaging microscopy (FLIM)-based monitoring of microglial metabolic alterations could be used to both differentiate surveillant microglia from activated microglia, as well as distinguish them from other CNS cell-types. Fluorescence lifetime is the measure of the average time a molecule remains in its excited state before returning to its ground state; a photon is emitted upon the return to ground state. FLIM measures the lifetime of each individual photon and creates a pixel contrast map based on the calculated exponential decay of each pixel.^19^ Importantly, unlike other fluorescence-based methods, fluorescence lifetime does not depend on concentration, absorption, or excitation intensity.^20^^–^^22^ Multiple physiologic parameters can change fluorescence lifetime including the local pH, ion, and oxygen concentrations, making FLIM an ideal method with which to image the cellular microenvironment. FLIM monitors metabolism by taking advantage of the intrinsic fluorescence of the ubiquitous metabolic coenzyme nicotinamide adenine dinucleotide (NADH) and flavin adenine dinucleotide (FAD). The fluorescence lifetime of protein-bound NADH changes depending on the enzyme to which it is bound.^23^ Recent evidence suggests that immune cells can switch from oxidative phosphorylation to aerobic glycolysis during inflammatory activation; much like the Warburg effect observed in tumor cells.^24^^–^^28^

Given the known metabolic changes that take place during microglial activation^29^ and the ability of FLIM to image free- and enzyme-bound NADH, we hypothesized that NADH lifetime and the fractional contribution of free NADH could be used to first distinguish microglia from other glial cell types, and then identify microglial activation status in the absence of additional fluorophore tagging. Previous efforts have exploited FLIM to examine the metabolic state of the cellular microenvironment and develop label-free signatures for different cell types ranging from bacteria to stem cells.^30^^,^^31^ A strategy reported by our group used FLIM as a means to noninvasively identify mammary tumor-associated macrophages in live mouse models.^32^ Another study also exploited the metabolic shift in macrophages to identify their phenotype label-free in cell culture.^33^

Here, we show that FLIM-based approaches can be used to observe metabolic changes in microglial cells using NADH lifetime signatures. We demonstrate the capability of FLIM to differentiate between surveillant and activated states of microglia both in primary mixed glial cultures and in tissue slices from adult mice treated with lipopolysaccharide (LPS). First, we tested different doses of LPS to examine changes in microglial FLIM signatures, then we tested the effect of different treatment times of a single LPS dose. We found that FLIM can be readily used to discriminate the activation status of microglia in tissue by quantifying NADH lifetime.

## 2 Materials and Methods

### 2.1 Animals

All animals were maintained in an AAALAC-accredited animal facility with a 12-h light/dark cycle regime and access to food and water ad libitum. All experiments were performed in accordance with protocols approved by the University of Wisconsin-Madison Institutional Animal Care and Use Committee and with the Guide to Care and Use of Laboratory Animals.

### 2.2 *In Vivo* Treatments

For FLIM imaging, 100-μm thick coronal slices were prepared from the fixed brains of 8-week-old, young adult C57BL/6J [wild type (WT)] mice (Jackson Labs, Bar Harbor, Maine). Mice were either given no treatment (control) or 5  mg/kg LPS (i.p. from *E. coli* O111:B4, Sigma) dissolved in sterile Hank’s balanced salt solution (HBSS), n=5 mice per treatment group. Three hours after LPS injection, animals were euthanized by isoflurane overdose and transcardially perfused with ice-cold phosphate-buffered saline (PBS), followed by a second perfusion with an ice-cold solution of 4% paraformaldehyde (PFA) in PBS. Brains were dissected, postfixed for 24 h in a solution of 4% PFA in PBS, and then moved to HBSS (all performed at 4°C and protected from light).

### 2.3 Preparation of Primary Neonatal Mixed Glial Cultures

Mixed primary glial cultures were prepared from 3-7A day old, CX3CR1-green-fluorescent protein (GFP) mouse pups as previously described.^34^ Briefly, brains were dissected immediately after decapitation, and the brain stem, cerebellum, olfactory bulbs, meninges, and visible blood vessels were removed. The remaining tissue was finely minced, thoroughly triturated with a serological pipette in 0.25% trypsin–ethylenediaminetetraacetic acid (EDTA) containing 0.1  mg/mL deoxyribonuclease I and then incubated at 37°C for 20 min. The reaction was immediately stopped by the addition of an equal volume of heat-inactivated horse serum. Dissociated cells were resuspended in Dulbecco’s modified Eagle’s medium supplemented with 10% fetal bovine serum and 100  units/mL penicillin/streptomycin. Brains were processed individually for each pup, and the resulting cell suspension was divided equally and plated in 35 mm dishes (one brain per four to six dishes). The plated cells were cultured for 7 to 14 days in a 37°C incubator supplemented with 5% ${\mathrm{CO}}_{2}$; the culture medium was replaced every 3 to 4 days.

### 2.4 Cell Culture Treatments

Mixed CX3CR1-GFP glial cultures were treated with HBSS (vehicle), 1, 10, or 100  ng/mL of LPS for 3, 8, or 24 h as indicated in the figure legends (n=5 independent biological replicates for each treatment). LPS is a ligand of the pattern recognition receptor TLR4 that mimics bacterial infection and activates inflammatory signaling pathways in microglia that lead to the production and release of inflammatory cytokines including IL-1β and TNFα. Preliminary experiments demonstrated no significant difference in mean NADH lifetimes in HBSS-treated cells between 3 and 24 h (data not shown). Following treatment, the culture medium was aspirated, and the cells were washed with cold HBSS before fixing with 4% PFA for 5 min, after which time the cells were washed again with cold HBSS and stored at 4°C protected from light.

### 2.5 Immunohistochemistry

100-μm thick coronal sections were prepared from the midbrain region of WT mouse brains (n=5 for each treatment group) using a Leica vibratome. Two slices from each animal were used for immunohistochemical staining. Briefly, slices were washed at room temperature with 0.3% TritonX-100 in PBS, before incubating in blocking buffer (1% bovine serum albumin, 0.3% TritonX-100/PBS) for 2 h at room temperature. To identify microglia, tissue slices were incubated with anti-Iba1 antibodies (1:1000; Wako Catalog No. 019-19741) in blocking buffer in the dark at 4°C overnight. Slices were washed again at room temperature with 0.3% TritonX-100 in PBS followed by incubation in the dark for 2 h with AlexaFlour594 anti-rabbit IgG antibodies (1:200; Thermo Fisher Scientific, Waltham, Massachusetts) in blocking buffer at room temperature. Slices were washed with 0.3% TritonX-100 in PBS and mounted on 1-mm slides using Cytoseal 60 (Richard-Allan Scientific, Kalamazoo, Michigan) mounting medium. Mounted sections were stored at room temperature, protected from light until imaging.

### 2.6 Multiphoton Lifetime Imaging

**Fig. 1:**
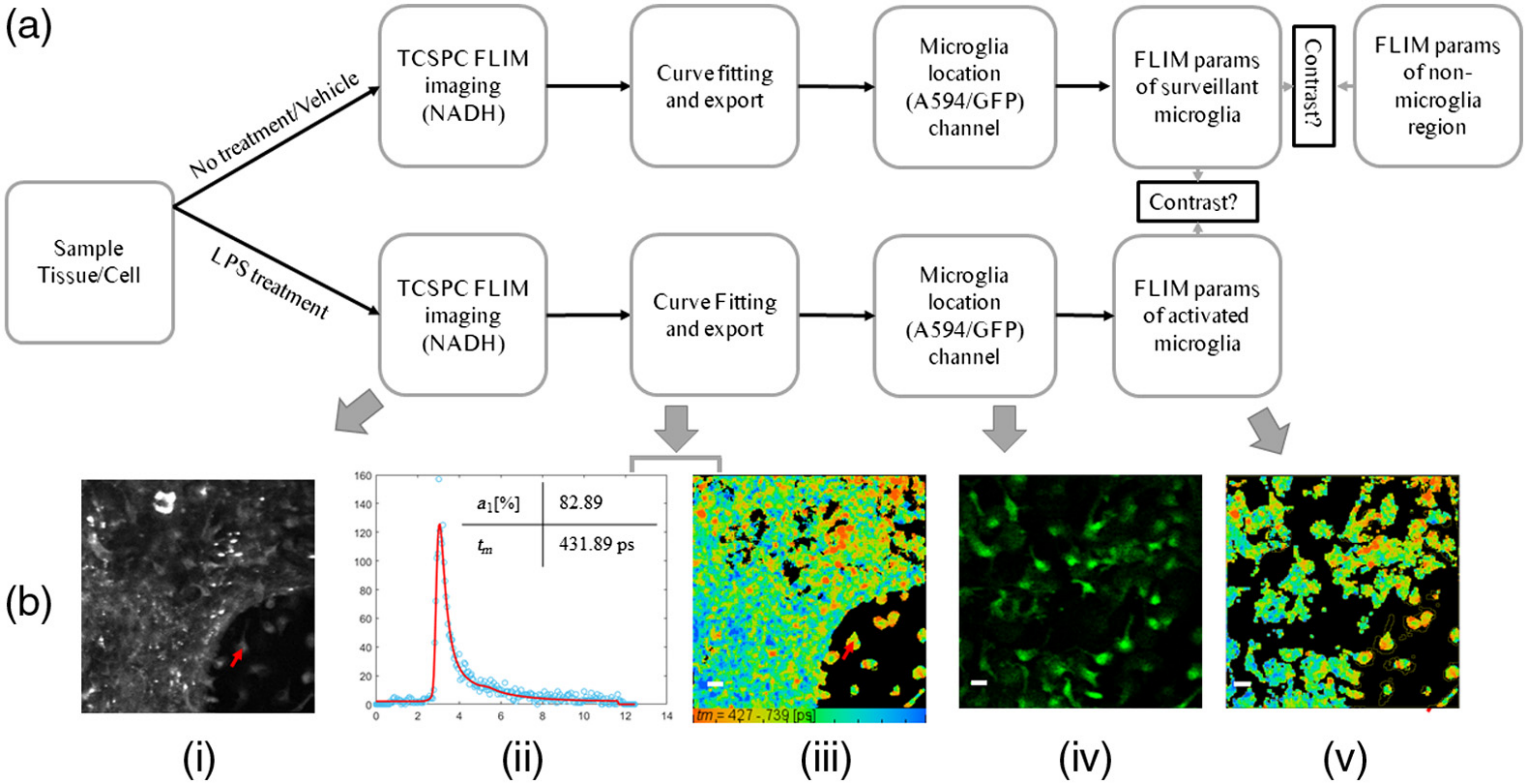
Methods to image and quantify microglia lifetime upon activation. (a) The steps for the imaging, data processing, and final comparison. A fluorescence intensity image was used to identify microglia in mixed glial cultures or brain tissue section. NADH FLIM parameters were extracted from both microglia and nonmicroglial regions. For activation experiments, only microglial FLIM parameters were compared. The example shown in (b) is a use case from a mixed glial culture representing each step outlined in (a). (i) An NADH intensity image of an FOV containing microglia. (ii) The lifetime decay curve of a single microglial pixel marked by the red arrow in (i). The table inset (ii) shows the mean lifetime ($t_{m}$) decay from two components (free and bound NADH) and the fractional contribution from free NADH (a1[%]). (iii) The fitted lifetime image for the FOV and the color bar show the range of lifetime in ps, (iv) the fluorescence (GFP or A594) intensity image was used to identify microglia and calculate average FLIM parameters. (v). Microglial lifetime image (Scale bar 20  μm).

Multiphoton lifetime^35^ and intensity imaging were performed on a custom multiphoton laser scanning system built around an inverted Nikon Eclipse TE2000U at the UW-Madison Laboratory for Optical and Computational Instrumentation.^36^ A 20× air objective (Nikon Plan Apo VC, 0.75 NA, Melville, New York, USA) was used for all imaging. For NADH imaging, data were collected using an excitation wavelength of 740 nm, and emission was filtered with a 457±50  nm bandpass filter (Semrock, Rochester, New York), corresponding to the spectral peak for NADH/NADPH (PH–phosphate). For GFP intensity imaging, excitation was set at 890 nm and a 520±35  nm bandpass emission filter was used (Semrock). For AlexaFluor594 imaging, excitation was set at 810 nm, and a 615/20 bandpass emission filter (Semrock) was used. For FAD imaging, excitation was set at 890 nm and a 520/35 bandpass emission filter was used (Semrock). FLIM images were collected at a 256×256  pixel resolution for 120 s using single photon counting (SPC)-150 Photon Counting Electronics (Becker & Hickl GbmH, Berlin, Germany) and a Hamamatsu H7422P-40 GaAsP photomultiplier tube (Hamamatsu Photonics, Bridgewater, New Jersey). The excitation power incident on cell/tissue was ∼12  mW for FLIM imaging. From a single dish or tissue section for each n, ∼20 neighboring field of views (FOVs) consisting of several hundred cells were randomly selected, and the mean signal was calculated based on the masking described in Sec. 2.6. The optical system instrument response function (IRF) was calibrated for each imaging session. Autofluorescence intensity and fluorescence lifetime data were analyzed in SPCImage (Becker & Hickl GmbH); a Levenberg–Marquardt routine for nonlinear fitting was used to fit the fluorescence decay curve collected for each decay after binning (factor 2). Data were assessed by the minimized chi-squared value generated during the fit so that the analysis was unbiased. To eliminate background fluorescence, a threshold was applied based on photon counts. A two-component fit was performed to account for free and bound NADH, and the mean lifetime was calculated with $\tau_{m}=a_{1}\tau_{1}+a_{2}\tau_{2}$, where $a_{1}$ and $a_{2}$ represent the fractional contribution from free and bound NADH, and $\tau_{1}$ and $\tau_{2}$ are lifetime constants of the free and bound components, respectively. For measuring the instrument response function, we used a urea crystal sample with 740-nm excitation and a 370/10 narrow bandpass (Semrock) filter to get the second harmonic generation signal. The use of a multiphoton laser scanning microscope modality for FLIM offers several advantages over confocal microscopy including deeper sample penetration, improved signal-to-noise ratios, enhanced sample viability (although all samples in the present studies were fixed), and it is tunable to multiple excitation wavelengths such as to image NADH, FAD, GFP, and Alexa 594. Figure 1 shows the experimental setup and image analysis

### 2.7 Data Analysis

The lifetime fitted data from SPCImage and all other parameters were exported into MATLAB^®^ (MathWorks Inc., Natick, Massachusetts) for calculating the means of custom regions and performing statistical analyses. The mean lifetimes and free/bound ratios were calculated for each sample. Repeated measures analysis of variance (ANOVA), paired sample t-tests were performed to determine the statistical significance in tissue culture experiments. A student’s t-test was performed for determining the significance in the tissue slice experiments from mice treated with LPS. Statistical significance was set at p<0.05. As mentioned previously, both the *ex vivo* and *in vitro* experiments consisted of five biological replicates per treatment. Each n was comprised of ∼20 neighboring FOVs per dish or tissue section, which were subsequently used to calculate average FLIM parameters for each treatment group.

## 3 Results

### 3.1 Microglial NADH Fluorescence Can Distinguish Reactive Microglia Following LPS Treatment

**Fig. 2:**
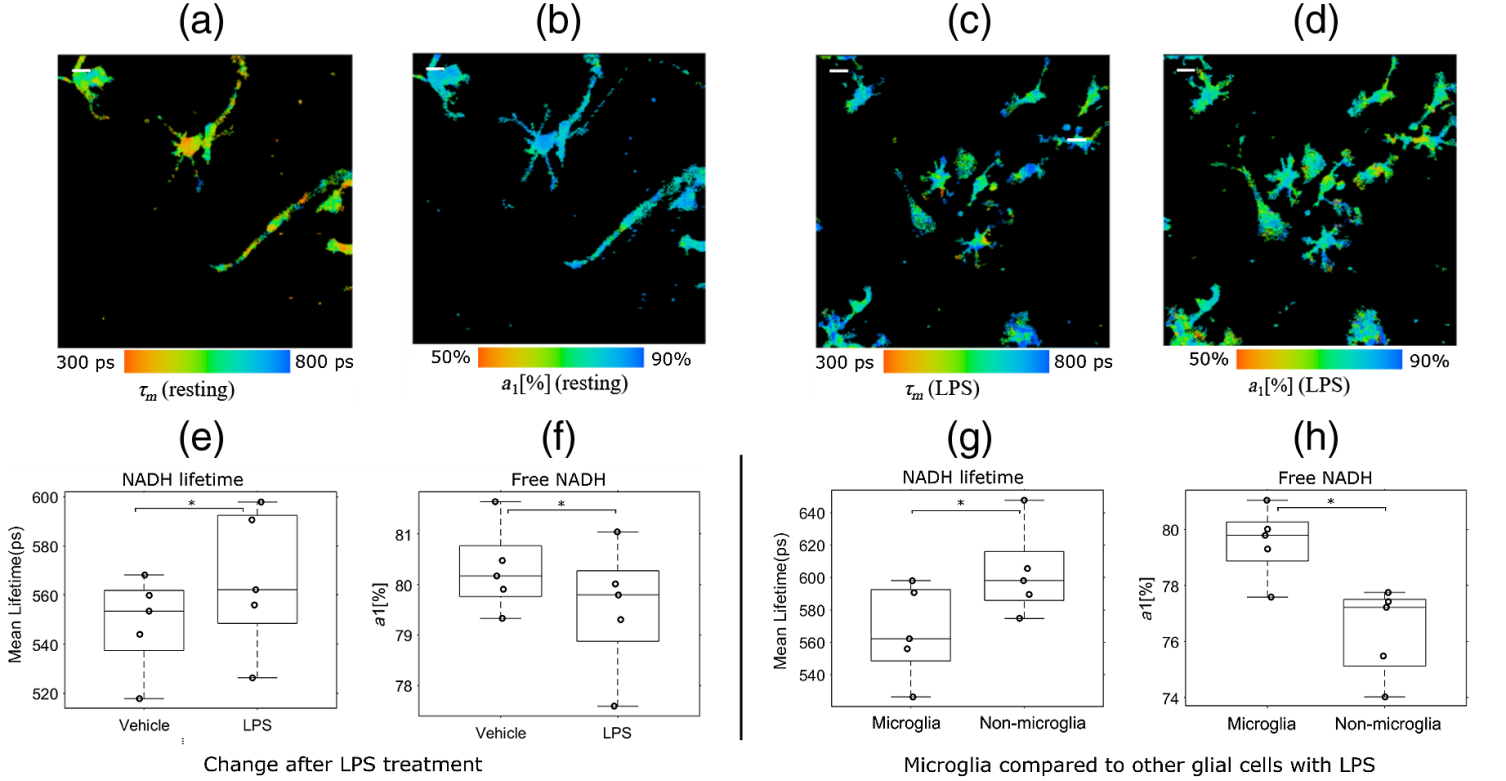
Relative change in microglia lifetime upon LPS treatment. Mixed glial cell cultures were treated with vehicle or LPS (10  ng/mL) for 1 h. Representative image of (a) NADH lifetime and (b) fractional free NADH from vehicle-treated microglia. Representative image of (c) NADH lifetime and (d) fractional free NADH from LPS-treated microglia. Quantification of (e) NADH lifetime and (f) free NADH levels in vehicle-treated microglia. Quantification of (g) NADH lifetime and (h) free NADH levels in LPS-treated microglia. LPS-stimulated microglia have a significantly higher mean lifetime and a lower free NADH contribution (n=5). Compared to nonmicroglia, microglia retain their lower lifetimes and higher free NADH levels even when exposed to LPS. T-test *p<0.05. (Scale bar 20  μm).

According to previous FLIM studies, a shorter mean lifetime and a higher fractional contribution implies greater free (and less bound) NADH.^37^^,^^38^ To study FLIM parameters in activated microglia, we investigated the relative change in NADH FLIM parameters in microglia stimulated with LPS.^39^^–^^42^ The underlying hypothesis of this experiment was that LPS treatment would initiate an inflammatory response resulting in a change in microglial metabolism, causing alterations in the metabolic cofactor NADH that could be assessed by FLIM. We treated mixed glial cultures with LPS (10  ng/mL) for 1 h. The mean NADH lifetime and fractional contribution of free NADH were compared between the vehicle- and LPS-treated groups. Representative images of microglial NADH lifetimes [Fig. 2(a) and 2(c)] and the fractional contributions of free NADH [Fig. 2(b) and 2(d)] from vehicle- and LPS-treated samples, respectively, show that LPS treatment significantly increased NADH lifetime (vehicle treatment 548.7±19.4  ps and LPS treatment 566.6±28.8  ps) in microglia [Fig. 2(e)]. In contrast, the free NADH fractional contribution [Fig. 2(f)] was significantly decreased (vehicle treatment 80.3%±0.85 and LPS treatment 79.5%±1.1). These data suggest that 1 h of LPS treatment can induce a rapid change in NADH lifetime, indicating that FLIM may be useful to monitor the status of microglial activation prior to a visible change in morphology. To confirm that microglia could still be distinguished from other glia in the context of LPS treatment, the FLIM parameters of microglia and nonmicroglial cells were compared. The mean microglia lifetime was significantly lower (566.6±28.8  ps) than nonmicroglia (603.1±27.4  ps) [Fig. 2(g)], whereas the fractional free NADH contribution in microglia was significantly higher (79.5%±1.1) than nonmicroglia (76.3%±1.5) [Fig. 2(h)].

**Fig. 3:**
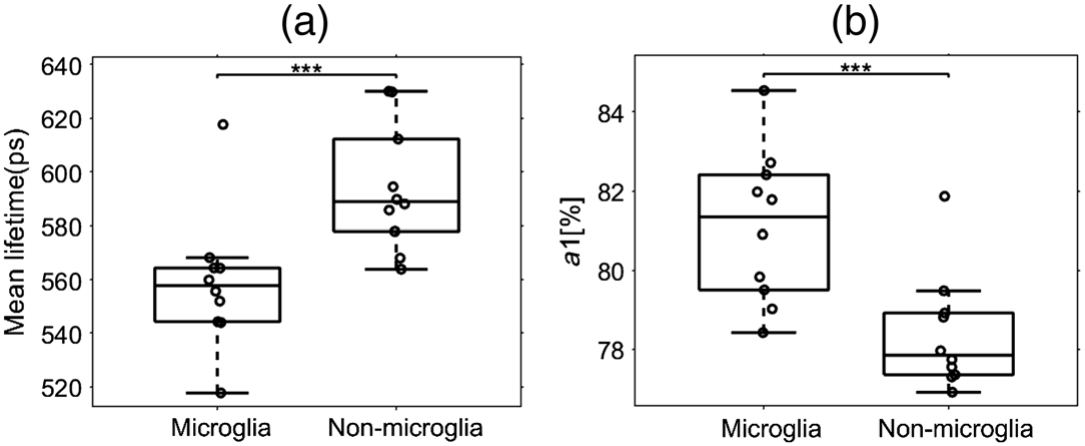
Comparison of microglia lifetime in mixed glial culture. (a) Microglia have a lower mean NADH lifetime and (b) higher free NADH levels than other cells in mixed glial cultures (n=10). T-test, ***p<0.005.

In addition, we compared the FLIM parameters of surveillant microglia with other cells in the mixed glial cultures. We looked at 10 different samples consisting of over a thousand cells from ∼20 different FOVs for each sample (Fig. 3). The mean lifetime for microglial cells was 558.8±25.4  picoseconds (ps), and the mean lifetime for nonmicroglia cells was 594.0±23.3  ps [Fig. 3(a)], whereas the fractional contribution from free NADH for microglia was 81.1%±1.8 and 78.4%±1.4 for nonmicroglia [Fig. 3(b)]. Together, these results indicate that microglia have a significantly lower mean NADH lifetime and a higher free NADH component compared to nonmicroglial cells.

### 3.2 FLIM Can Be Used to Characterize Microglia in Brain Tissue

**Fig. 4:**
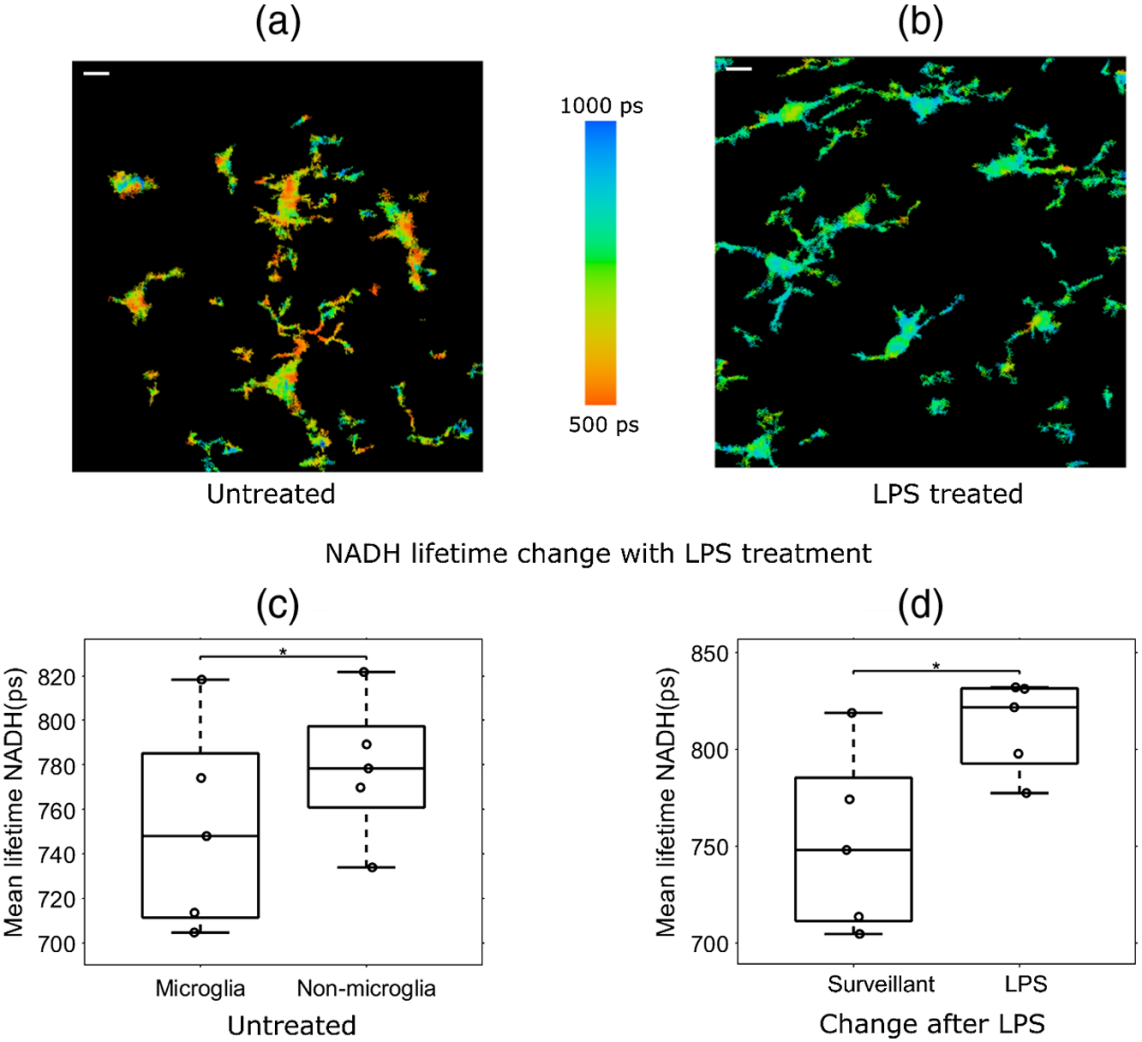
NADH lifetime can be used to distinguish microglia from nonmicroglia and characterize microglial activation status in brain tissue. A microglial mask was created using the AlexaFluor594 intensity image from Iba1-labeled brain tissue slices from WT mice that were untreated or treated with LPS (5  mg/kg). Representative image of microglial NADH lifetime in tissue slices from (a) vehicle- or (b) LPS-treated mice (n=5). (c) Microglia had a significantly shorter NADH lifetime than nonmicroglial cells in tissues from both vehicle treatment. (d) The lifetime of NADH increases in mice after LPS treatment. t-test, p<0.05. (Scale bar 10  μm).

After establishing that FLIM parameters can be used to characterize microglia in mixed glial cell cultures, and that FLIM can distinguish activated microglia, we next extended our observations to test whether this trend holds in intact brain tissue. Brain tissue was sectioned for *ex vivo* multiphoton FLIM imaging from WT mice that were either given no treatment or treated *in vivo* with LPS (5  mg/kg) for 3 h. The goals of this experiment were twofold: (1) to investigate whether microglia have a unique lifetime signature in relation to other brain cell types in tissue and (2) to determine if microglial activation causes alterations in FLIM NADH lifetime and free NADH level parameters in tissue. Microglia were labeled with anti-Iba1 antibodies, followed by an Alexa Fluor 594 secondary antibody. Representative NADH lifetime images of microglia from untreated [Fig. 4(a)] and LPS-treated [Fig. 4(b)] mice showed a statistically significant reduction in microglial NADH mean lifetime (751.6±46.58  ps) compared to nonmicroglial cells (778.6±28.2  ps) in the tissue [Fig. 4(c)]. As we had observed in mixed glial cultures, we found that microglia had a longer mean NADH lifetime (811.8±23.7  ps) in tissue slices from LPS-treated mice compared to microglia from untreated mice (751.6±46.58  ps) [Fig. 4(d)]. Furthermore, we found that LPS treatment increased lifetime from 668.42±34.48  ps to 683.16±41.35  ps in a GFP mice. In addition, we observed similar LPS-induced shifts in lifetime values in WT tissue slices stained with Iba1 (not shown) suggesting that GFP expression does not interfere with relative lifetime changes.

### 3.3 LPS Dose Dependently Alters NADH Lifetime and Free NADH Ratio

**Fig. 5:**
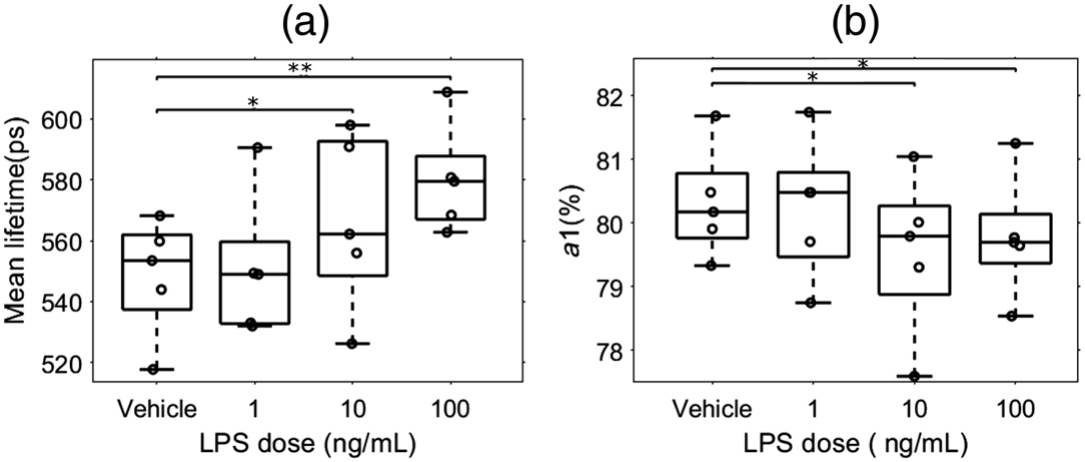
Microglia FLIM parameters vary with LPS dose. LPS dose dependently alters NADH lifetime and free NADH ratio. Mixed glial cell cultures from CX3CR1-GFP mice were treated with vehicle, 1, 10, or 100  ng/mL of LPS for 1 h. (a) NADH lifetime and (b) free NADH levels were measured in ${\mathrm{GFP}}^{+}$ cells. Compared to vehicle treatment, the 10 and 100  ng/mL doses significantly increased NADH lifetime and decreased free NADH levels. One-way ANOVA, *p<0.05, **p<0.01.

We next sought to determine whether LPS treatment could dose dependently alter microglial NADH lifetime and free NADH levels. To test this, we treated mixed glial cultures with 1, 10, and 100  ng/mL LPS. We found that NADH lifetimes were indeed dose dependently increased by LPS treatment (Fig. 5). While the NADH lifetime of cells treated with vehicle (548.4±19.1ps) did not differ from 1  ng/mL LPS (540.2±34.3  ps), microglial lifetimes in 10  ng/mL (568.4±28.5  ps) and 100  ng/mL (578.4±23.5  ps) LPS were significantly increased over control treatment, as expected [Fig. 5(a)]. The fractional NADH contribution (a1) in contrast, dose dependently decreased with increasing LPS concentrations [Fig. 5(b)]. Again, whereas free NADH levels between vehicle (80.3%±0.9) and 1  ng/mL LPS (80.2%±1.1) did not differ, they were significantly decreased in the 10  ng/mL (79.2%±1.2) and 100  ng/mL (79.8%±1.1) LPS doses.

### 3.4 LPS Treatment Time Dependently Increases Microglial NADH Lifetime

**Fig. 6:**
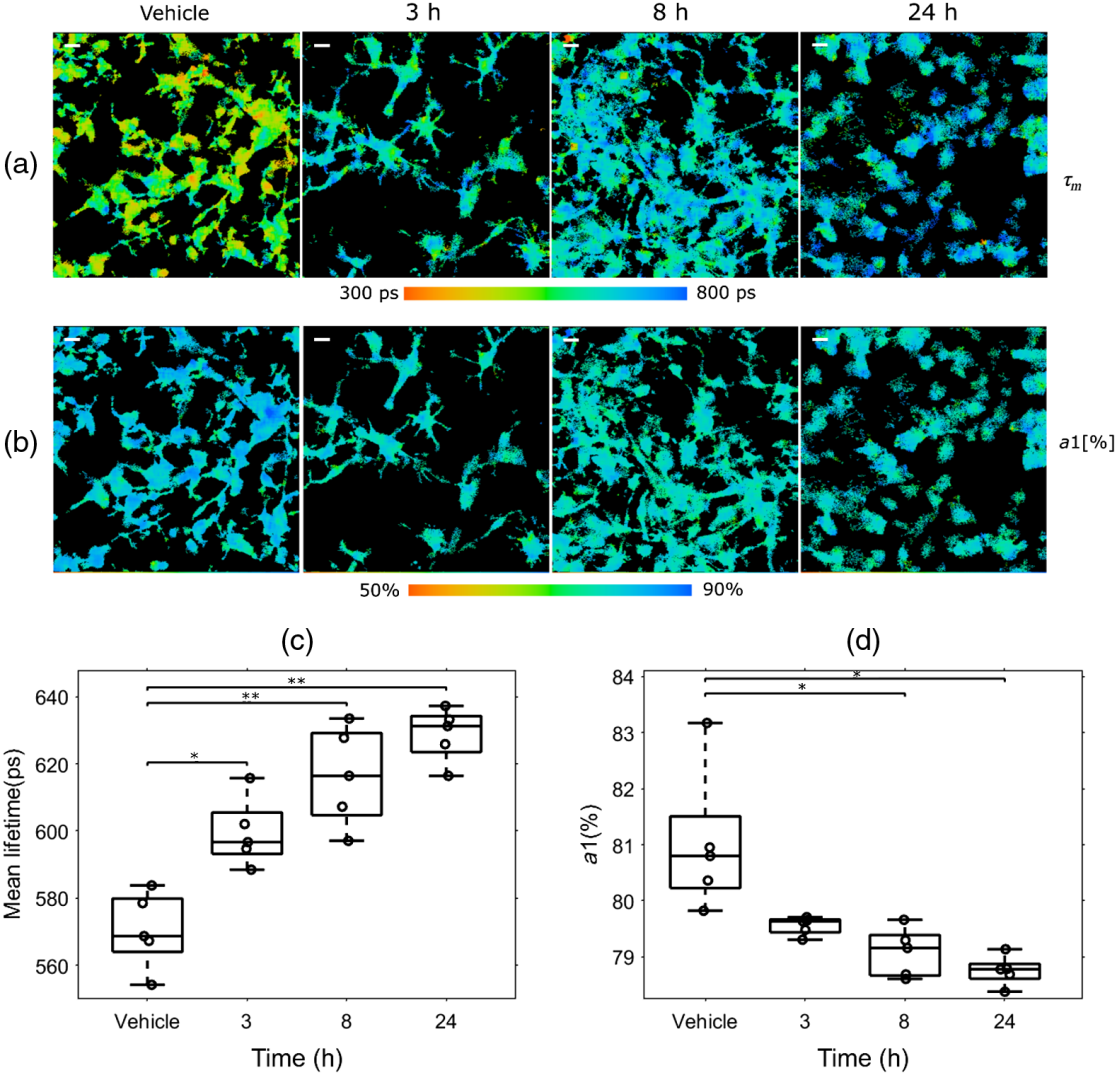
NADH lifetime dose dependently increases with time post-LPS treatment. Mixed glial cultures from CX3CR1-GFP mice were treated with vehicle or 10  ng/mL LPS for 3, 8, or 24 h (n=5). (a) Representative NADH lifetime and (b) fractional free NADH levels images from microglia are shown. Quantification of (c) NADH lifetime and (d) free NADH levels in LPS-treated microglia show time-dependent changes in FLIM parameters. The mean microglial NADH lifetime increases as early as 3 h after LPS exposure; free NADH levels significantly decrease within 8 h of LPS exposure. One-way ANOVA *p<0.05, **p<0.01. (Scale bar 20  μm).

To determine whether the observed differences in FLIM parameters changed with time post-LPS treatment, mixed glial cultures were treated with 10  ng/mL LPS for 3, 8, and 24 h. We chose the 10-ng/mL dose of LPS because it was the lowest dose tested that had statistically significant effects on FLIM parameters. Representative images of the mean microglial NADH lifetime [Fig. 6(a)] and the fractional contribution from free NADH [Fig. 6(b)] are shown. LPS significantly increased the NADH lifetime of microglia at 3 h (599.5±10.2  ps), 8 h (616.4±14.7  ps), and 24 h (628±8  ps) compared to vehicle-treated cells (570.5±11.3) ps [Fig. 6(c)]. The fractional free NADH components significantly decreased from vehicle treatment (80.9±1.3%) at both 8 h (79.01±0.4%) and 24 h (78.7±0.2%), but not at 3 h (79.5±0.2%) [Fig. 6(d)].

## 4 Discussion

Here, we report a method of differentiating microglial activation in relation to surveillant microglia using FLIM measurements of the intrinsically fluorescent metabolic cofactor NADH. We show that NADH FLIM can be used to characterize the activation state of microglia both *in vitro* and *ex vivo* in brain tissue, and that LPS dose and treatment time directly impact FLIM parameters. In addition, we found that microglia have different FLIM parameter values in relation to other glial cells in mixed glial cultures and in brain tissue sections. Based on previous studies, a relative change in NADH lifetime reflects a change in metabolic state of the intracellular microenvironment such that a lower mean lifetime implies greater free NADH.^37^^,^^38^ We found that microglia in their surveillant or unactivated state had a shorter NADH mean lifetime compared to other glial cells, and that they had a greater fractional contribution from free NADH. In contrast, LPS treatment increased their mean NADH lifetime and decreased the fractional contribution of free NADH (compared to vehicle treatment). To our knowledge, this is the first study to characterize microglia and to study the glial response to an inflammatory stimulus using fluorescence lifetime imaging.

In brain tissue slices, we found that microglia had a lower mean NADH lifetime than other cells in the tissue and that LPS treatment increased their mean lifetime for NADH, supporting our contention that NADH lifetime can be used as a signature to differentiate between surveillant and activated microglia in the brain. In the mixed glial culture, there was a decrease in the fractional contribution from NADH in tissue after 3 h of LPS treatment, although it did not quite reach statistical significance (p=0.058). There was, however, a statistically significant increase in the NADH mean lifetime. But in tissue, differences in microglial NADH FLIM were observed within 1 h of LPS treatment even before visible morphological changes appeared. Based on these observations, we postulate that mean NADH lifetime is well-suited for identifying a microglial fingerprint upon activation. FAD being the principle electron donor in the electron transport chain is an important metabolic cofactor in the cellular respiratory process and together with NADH intensity can be used to determine the redox state of the cell.^43^^–^^46^ Based on the metabolic state, the FAD lifetime and relative free/bound state also change. Therefore, FAD lifetime may also be useful as an additional parameter to NADH lifetime. In tissue slices, we chose Alexa 594 as the fluorophore to identify microglia. This freed the FAD emission channel allowing us to test the efficacy of the FLIM technique with a microglial marker independent of GFP fluorescence. We found that FAD lifetime had similar changes to NADH lifetime both when compared to nonmicroglial cells and microglial activation status (Fig. S1 in the Supplementary Material). Because of the relative weakness of FAD intensity and overlap with other fluorophore spectra, FAD FLIM imaging can be challenging. In future work, FAD lifetime changes need to be quantified in both dose and time variation studies to see whether the trend holds like NADH. Future studies may also exploit both NADH and FAD lifetime changes simultaneously to provide better quantification of the microglial redox state and indicate their activation status. We would also like to draw attention to the trend of change in lifetime and free NADH ratio. According to the definition of mean lifetime described in Sec. 2, the mean lifetime is dependent on free NADH. Therefore, the changes have similar patterns. But they might not always necessarily result in the same conclusion. For example, in Sec. 3.4, the 3-h time point showed a significant change in lifetime, but not the free NADH ratio. In cases where free and bound lifetime had significant alterations, the trend might not be as similar, so probing both parameters in the future might be helpful to better understand the changes.

Our observation that NADH lifetime increases upon LPS treatment suggests that microglia become more oxidative upon inflammatory activation. Although there is evidence that reparative macrophages tend to be more oxidative rather than glycolytic,^33^^,^^47^^–^^49^ the metabolic status of LPS-stimulated microglia needs to be verified in a separate experiment. It is important to note that the lifetime changes observed here following LPS activation may not necessarily be the same as microglial inflammatory activation by other stimuli, such as aging, neurodegenerative disease, or sleep deprivation/fragmentation disorders. Additional studies are needed to quantify FLIM parameters in the context of these scenarios since FLIM may be extended to study the behavior of microglia in aging,^34^ multiple sclerosis, and neurodegenerative diseases, such as Alzheimer’s and Parkinson’s diseases.

One caveat to note in these studies is that FLIM parameters may be altered in mixed glial cell cultures due to the extraction and culture process. However, since we noted similar changes in brain tissue after *in vivo* LPS treatment, it is unlikely that the altered FLIM signals we have noted are an artifact of the *in vitro* culture system. There may also be concern about lifetime shifts due simply to the fixation process. In this regard, our lab recently demonstrated that fixation does not change the metabolic contrast.^50^ Although the absolute lifetime values may be shifted by fixation, the treatment-induced alterations should be relative. In addition, the accuracy of the fit will depend on the signal-to-noise ratio. Thus, it is likely that a sample with a low signal-to-noise ratio, the fit and overall result might be inaccurate. Finally, it is important to note that recent work^51^ showed possible overlap between GFP and NADH spectra, which may confound interpretation of FLIM results. While this will require further investigation, when we compared lifetime pattern shifts in WT and CX3CR1-GFP brain tissue slices, we found that NADH lifetime was still increased upon LPS treatment, regardless of the presence of GFP (data not shown). Likewise, we saw similar FLIM signature and activation trends in brain tissue using A594, which is well separated from the NADH spectra. The increase in NADH lifetime as a result of LPS-induced activation would suggest a more oxidative nature of active microglia compared to surveillant; however, further experiments would need to be done to confirm their metabolic state and the resulting shift in lifetime. Finally, although there may be variability among FLIM systems based on the optics and electronics used, the trends and relative changes in lifetime should be comparable. FLIM can also be used to measure the variability within a specific system.

In the future, NADH and FAD FLIM parameters may be used to determine whether microglia and infiltrating macrophages can be distinguished using cell-specific markers and advanced computational techniques (such as machine learning). Another important direction for future studies would be to utilize FLIM to differentiate microglial phenotypes^52^ to better understand their transition between different immune activities and test the effectiveness of therapeutic drugs.^53^ A major limitation for using FLIM assessments in tissues or *in vivo* is the complexity of distinguishing microglial signatures in the context of multiple cell populations and tissue environments. While work is ongoing to improve the speed and accuracy of FLIM for complex multicomponent measurements, FLIM by itself may be insufficient in some cases. Accordingly, we are exploring machine learning-based approaches to exploit the NADH/FAD lifetime differences in microglia compared to the surrounding tissue in metabolically active situations, such as *in vivo*.

## 5 Conclusion

Using a multiphoton FLIM-based technique, we have characterized microglia and their activation status. Our results demonstrate that LPS-activated microglia have a higher NADH lifetime than surveillant microglia. We found that the change in NADH FLIM parameters of activated microglia was both LPS dose and treatment time dependent, which may be indicative of their activation state. In the future, the relative increase in NADH lifetime may be used to characterize cell activation in various neurodegenerative disorders in the CNS. We conclude that FLIM can be a powerful tool with which to monitor changes in microglial activation states or metabolic changes, perhaps in the absence of additional immunostaining.
