# Supplementary material for: Microglia activation visualization via fluorescence lifetime imaging microscopy of intrinsically fluorescent metabolic cofactors
Source: Neurophotonics. 2020 Aug 8;7(3):035003. doi: 10.1117/1.NPh.7.3.035003 (PMC7414793; doi:10.1117/1.NPh.7.3.035003)
Supplement: Supplementary file 1 [file NPh_007_035003_SD001.docx]

**Microglia activation visualization via Fluorescence lifetime Imaging microscopy of intrinsic metabolite**

**Md Abdul Kader Sagar^1^, Jonathan N Ouellette^2^, Kevin P Cheng^1^, Justin C Williams^1^, Jyoti J Watters^2#^, Kevin W Eliceiri1,^3,4#^**


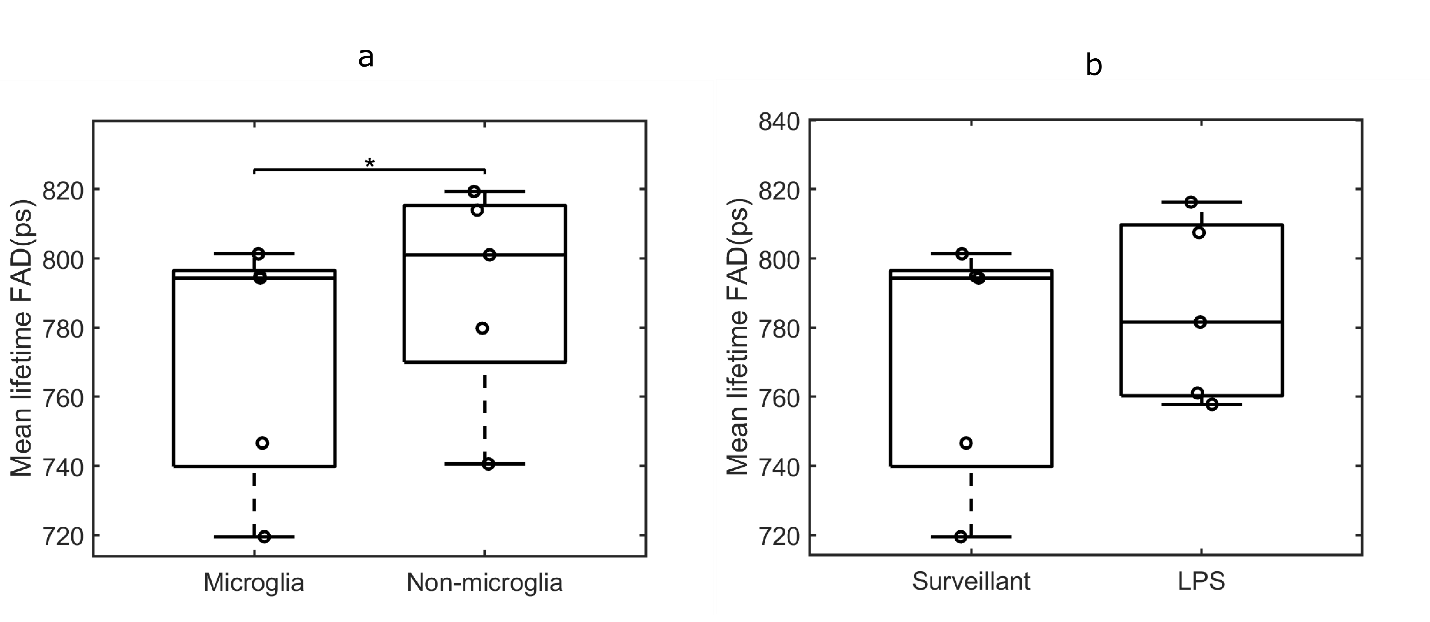


**Supplementary figure 1: FAD lifetime can distinguish microglia in tissue from mice treated with LPS.** A microglial mask was created using the A594 intensity image from Iba1-labeled brain tissue slices from untreated or LPS-treated (5mg/kg) WT mice for 3 hrs. Microglial (a) FAD lifetime is lower than in non-microglial cells. (b) LPS treatment increases FAD lifetime, but the change was statistically insignificant (n=5, t-test, p = 0.17).


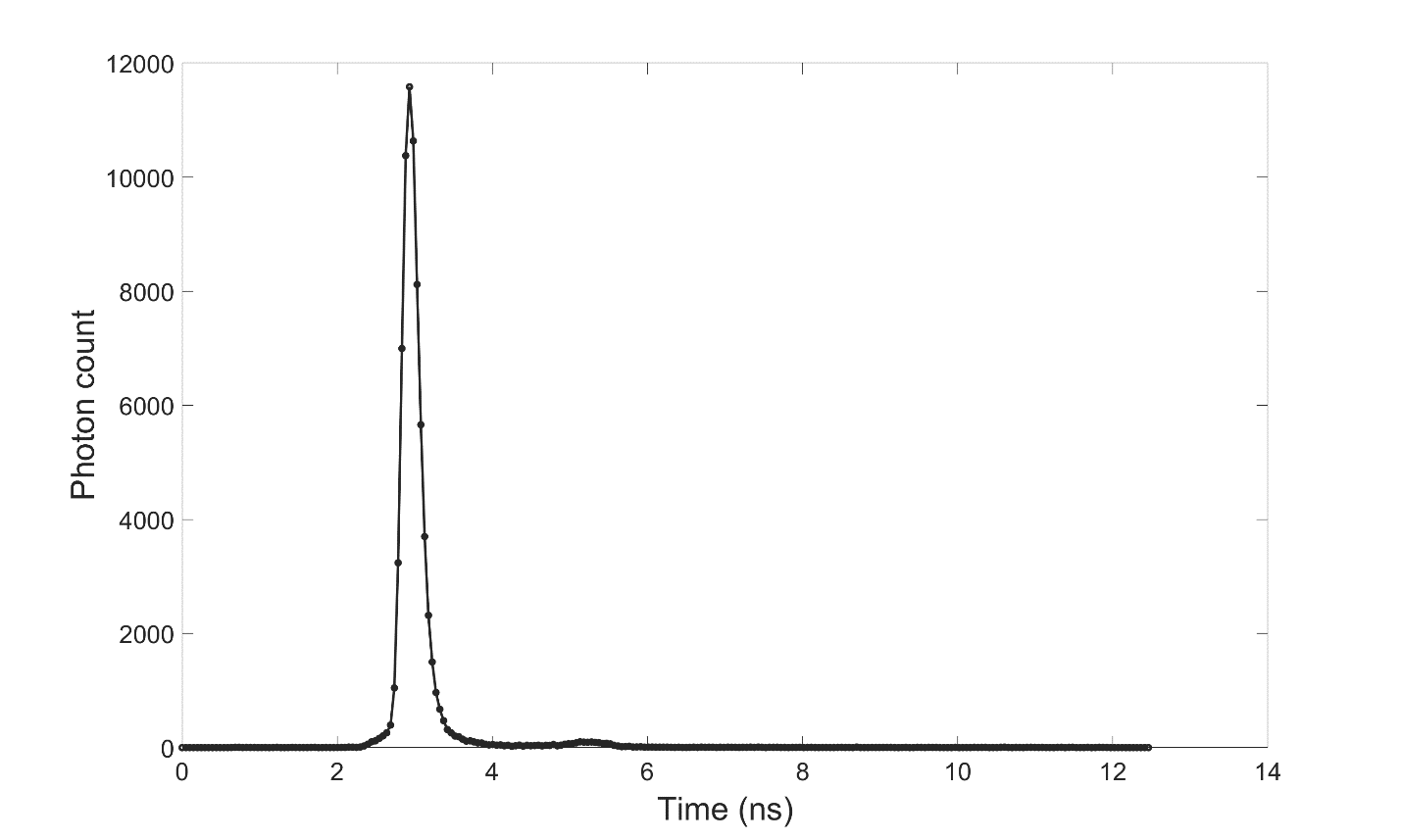


**Supplementary figure 2:** Instrument response function (IRF) used for the analysis


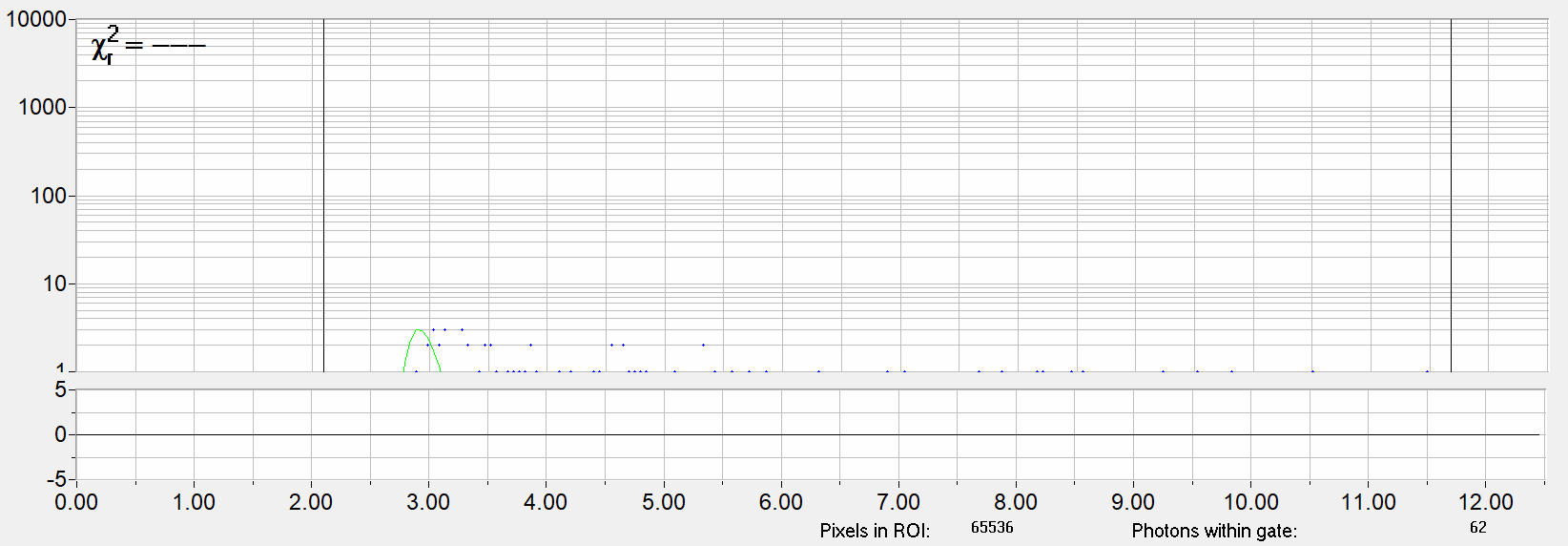


**Supplementary figure 3:** Sample decay from a region without sample
